# MicroRNA-141-3p promotes glioma cell growth and temozolomide resistance by directly targeting p53

**DOI:** 10.18632/oncotarget.20528

**Published:** 2017-08-24

**Authors:** Xu Zhou, Weining Wu, Ailiang Zeng, Er Nie, Xin Jin, Tianfu Yu, Tongle Zhi, Kuan Jiang, Yingyi Wang, Junxia Zhang, Yongping You

**Affiliations:** ^1^ Department of Neurosurgery, The First Affiliated Hospital of Nanjing Medical University, Nanjing, Jiangsu 210000, China

**Keywords:** glioblastoma, proliferation, TMZ resistance, microRNA, p53

## Abstract

Glioblastoma multiforme is the most common primary malignancy in the brain and confers a uniformly poor prognosis. MicroRNAs have been shown to activate or inhibit tumorigenesis. Abnormalities in the p53 signaling pathway are found in various cancers and correlate with tumor formation. We examined the expression of microRNA-141-3p (miR-141-3p) in glioma of different grades by analysis of expression profiling databases and clinical specimens. Cell proliferation and flow cytometry assays were performed to evaluate the promotion of miR-141-3p in proliferation, cell cycle, apoptosis, and temozolomide resistance of glioblastoma cells *in vitro*. Bioinformatics analyses, luciferase reporter assays, and immunoblotting showed that p53 is a target gene of miR-141-3p. A significant inverse correlation was observed between expression of miR-141-3p and p53 in glioma and normal brain tissues (R^2^=0.506, P<0.0001). Rescue experiments indicated that overexpression of p53 significantly reversed the alterations in proliferation, cell cycle distribution, and temozolomide resistance measured by cell apoptosis induced by miR-141-3p overexpression. In an orthotopic mouse model of human glioma, inhibition of miRNA-141-3p reduced the proliferation and growth of glioma cells in the brain and significantly prolonged the survival of glioma-bearing mice. We suggest that miR-141-3p promotes glioblastoma progression and temozolomide resistance by altering p53 expression and therefore may serve as a new diagnostic marker and therapeutic target for glioma in the future.

## INTRODUCTION

Glioblastoma multiforme (GBM) is the most common type of primary brain tumor and has shown the highest mortality rate among all brain malignancies during the past 40 years [1]. Approximately 20,000 new cases of glioma are diagnosed in the United States every year [2]. Despite treatment with surgery, chemotherapy, and/or radiotherapy, the median survival of patients with GBM, the most aggressive type of malignant glioma, is only 12 to 14 months [3]. Therefore, it is necessary to investigate the mechanism involved in the development and progression of glioma and find a more effective therapy. Temozolomide (TMZ), a DNA alkylating antineoplastic drug, is a promising chemotherapeutic agent that readily crosses the blood–brain barrier and is used as first-line therapy for the treatment of glioblastoma [4]. It can efficiently inhibit the proliferation of glioma cells and induce apoptosis [5, 6]; however, a major impediment to effective treatment is acquired resistance to TMZ. Thus, further studies that aim to enhance the therapeutic effect of chemotherapy drugs are essential.

MicroRNAs (miRNAs), a series of small, highly conserved, non-coding RNA molecules 18–25 nucleotides in length, are known to activate or inhibit the progression of various cancers, including glioma, and have been proposed as novel targets for anticancer therapies in recent years [7, 8]. More than 50% of miRNAs are known to be involved in human tumorigenesis by directly targeting oncogenes or tumor suppressor genes [9]. For instance, miR-21, one of the first miRNAs detected in the human genome, accelerates tumorigenesis by targeting several tumor suppressor genes [10, 11]. We have reported that miR-92b expression correlates with glioma WHO grade and promotes glioma proliferation and invasion by targeting beta-catenin [12]. However, it is likely that there are other unidentified grade-associated miRNAs that also activate or inhibit tumorigenesis in glioma.

In conclusion, temozolomide (TMZ) is the first-line therapy for the treatment of glioblastoma, miRNA has been studied as a new way for glioma treatment. Based on this, we want to find a novel miRNA that can treat temozolomide resistance-glioma more effectively.

More than 30 years of research on tumor suppressor p53 have yielded over 50,000 publications, making p53 one of the most studied proteins in science and underlining its significance for understanding the mechanisms of GBM [13]. When it was discovered in 1979, p53 was originally thought to be an oncogene because of its mutated cDNA and high expression in various cancers [14, 15]. In fact, inhibition of p53 activates the formation of cancers, with a well-established impact on the cell cycle and apoptosis [16, 17]. The cyclin-dependent kinase inhibitor p21, which is important in regulating cell cycle progression, and the apoptosis promoter bax, which is vital for cell apoptosis, are also induced by p53 [18]. Furthermore, in recent study, p53 has been found to sensitize temozolomide treatment in glioblastoma [19].

In the present study, we showed that the oncogenic miR-141-3p was increased in clinical GBM samples and correlated with WHO grade. Moreover, we identified p53 as a direct target for miR-141-3p, and showed that p53 was decreased in GBM with a negative correlation between miR-141-3p and p53 level. We also found that miR-141-3p activated proliferation, cell cycle regulation, cell apoptosis, and TMZ resistance by targeting p53 in wild-type p53 glioblastoma cells. Our findings suggest that miR-141-3p could be a critical therapeutic target for GBM intervention.

## RESULTS

### MiR-141-3p is increased in human glioma tissues

**Figure 1:**
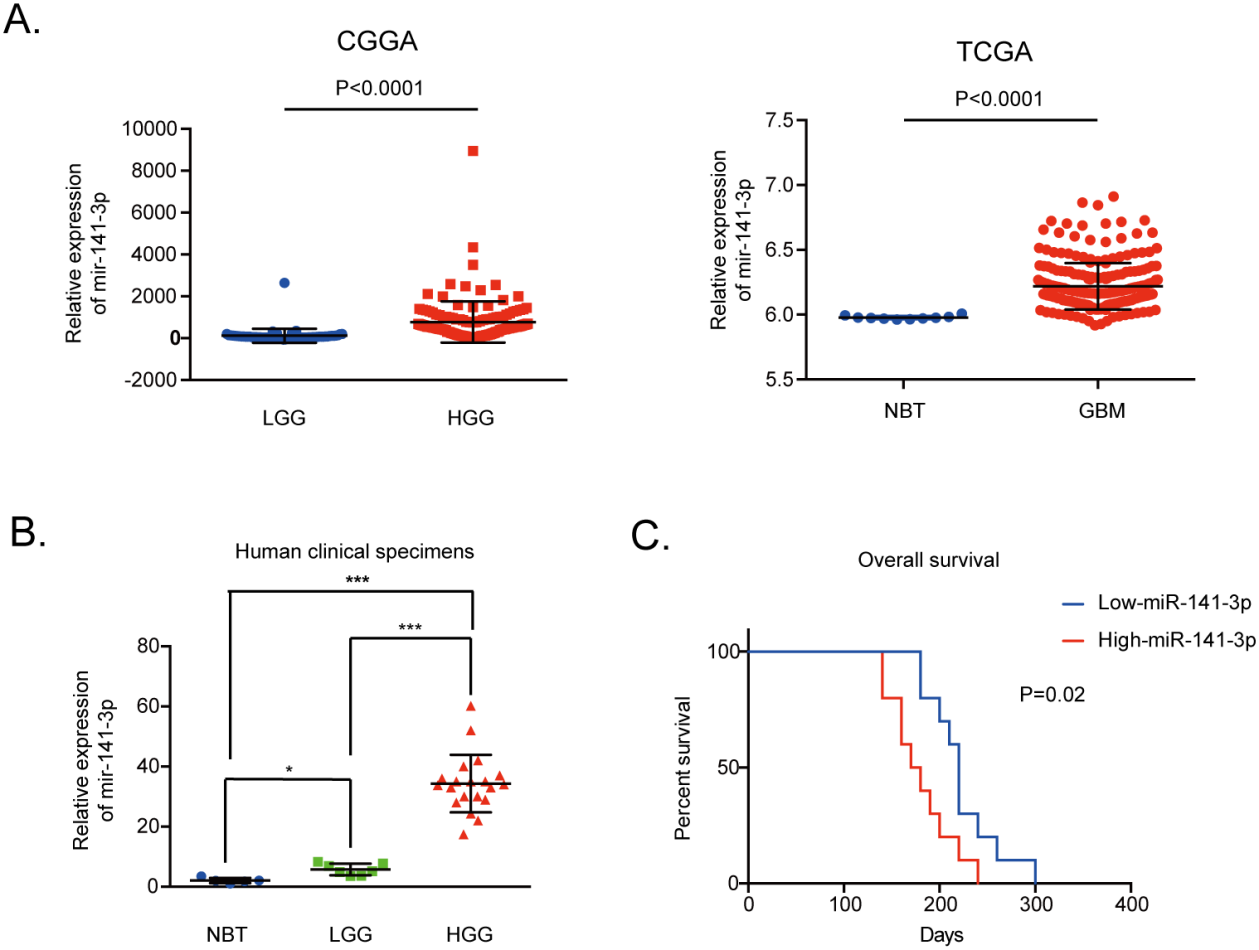
MiR-141-3p expression correlates positively with malignant degrees of glioma **(A)** Expression of miR-141-3p positively correlates with the WHO grade in the CGGA Public database, non-cancerous brain and GBM specimens in the TCGA Public database. **(B)** Relative levels of miR-141-3p in five non-cancerous brain specimens, seven low-grade glioma specimens, and 20 high-grade glioma specimens. **(C)** Correlation between miR-141-3p expression and the overall survival of GBM patients analyzed by Kaplan-Meier survival curves. A log-rank test was used to assess the statistical significance of differences. Results are presented as the mean ± S.D. *P<0.05, **P<0.01, and ***P<0.001.

We evaluated the expression level of miR-141-3p in 158 glioma tissues of different grades from the CGGA database and 255 glioma specimens of non-cancerous brain and GBM in the TCGA Public database. The data indicated that the expression level of miR-141-3p was significantly higher in high-grade glioma (grade III and IV) tissues compared with low-grade glioma (grades I and II) tissues from the CGGA database as well as higher in GBM compared to non-cancerous brain in the TCGA Public database (P<0.0001; Figure 1A). Moreover, for clinical human glioblastoma specimens, miR-141-3p expression levels were markedly higher in the 20 high-grade glioma tissues than in five non-cancerous brain tissues or seven low-grade glioma tissues (Figure 1B). Analysis of the Kaplan–Meier survival curve for 20 cases of clinical GBM and 64 GBM tissues from the CGGA database showed that high expression of miR-141-3p (classified as higher than the medium value) was associated with decreased survival relative to those with low miR-141-3p levels (P=0.02, Figure 1C; P=0.0207, Supplementary Figure 4A).

### MiR-141-3p activates glioma cell growth *in vitro*

**Figure 2:**
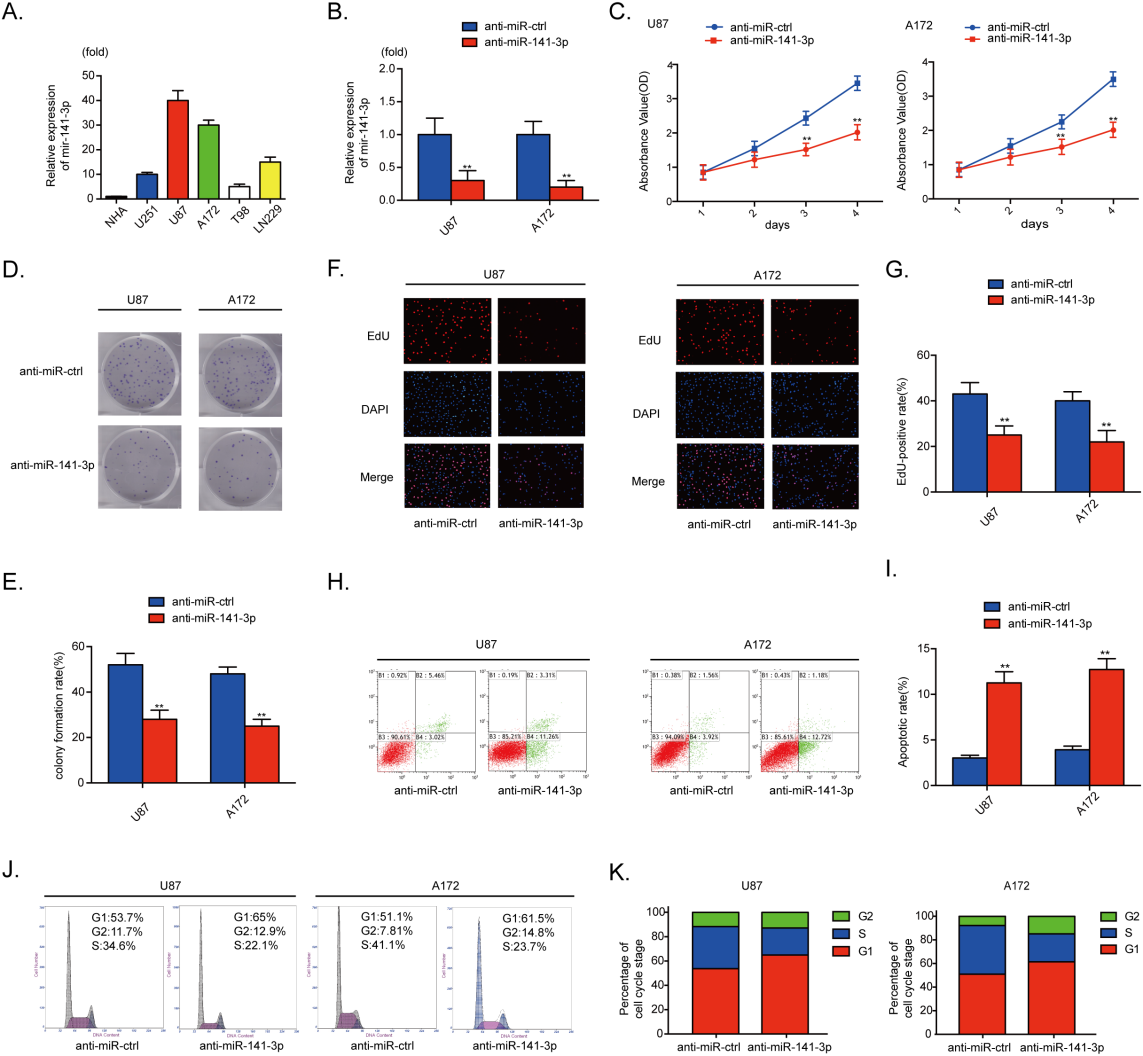
MiR-141-3p promotes glioma growth **(A)** Relative expressions of miR-141-3p in glioma cell lines A172, U87, U251, T98, and Ln229 and normal human astrocytes. **(B)** Relative expression of miR-141-3p in U87 and A172 cells was analyzed by qRT-PCR after transfection. **(C)** Cell proliferation was detected by CCK8 assays in U87 and A172 cells transfected with anti-miR-ctrl or anti-miR-141-3p. **(D** and **E)** Colony formation assays in U87 and A172 cells transfected with anti-miR-ctrl or anti-miR-141-3p. Scale bar: 500 μm. **(F** and **G)** Respective merged images of U87 and A172 cells in EDU transfected with anti-miR-ctrl or anti-miR-141-3p after 48 h. Representative images are shown (original magnification, 200×). **(H** and **I)** Apoptosis rates of U87 and A172 cells transfected with anti-miR-ctrl or anti-miR-141-3p after 48 h were detected by flow cytometry. All experiments were performed three times using triplicate samples. Average values are indicated with error bars in the histogram. **(J** and **K)** The cell cycle of U87 and A172 cells transfected with anti-miR-ctrl or anti-miR-141-3p after 48 h was detected by flow cytometry. All experiments were performed three times using triplicate samples. Average values are indicated with error bars in the histogram. Results are presented as the mean ± S.D. *P<0.05, **P<0.01, and ***P<0.001.

The relative levels of miR-141-3p in various glioma cell lines (A172, U87, U251, T98, Ln229) and normal human astrocytes were analyzed by qRT-PCR (Figure 2A). To examine the biological significance of miR-141-3p in glioma, anti-miR-141-3p or anti-miR-ctrl was transiently transfected into A172 and U87 cells that express high levels of miR-141-3p. qRT-PCR results showed that the relative expression level of miR-141-3p in U87 cells and A172 cells treated with anti-miR-141-3p was significantly reduced compared with cells treated with anti-miR-ctrl (P<0.01 for both cell lines; Figure 2B). Inhibition of miR-141-3p had remarkable inhibitory effects on cell viability in U87 and A172 cells compared with the control group by CCK-8 assay and colony formation assay (Figure 2C-2E). An EDU cell imaging assay was performed to further evaluate the effect of miR-141-3p on proliferation (Figure 2F) and yielded results similar to those of CCK-8 and colony formation assays. The EDU-positive rate in U87 and A172 cells transfected with anti-miR-141-3p was lower than that of the control group (P<0.01, Figure 2G), indicating that the proliferation of glioma cells was suppressed by inhibition of miR-141-3p. The cell apoptosis rate and cell cycle distribution of cells with knockdown of miR-141-3p was also investigated. Flow cytometric analysis showed that knockdown of miR-141-3p promoted cell apoptosis, increased the apoptosis rate, induced G1/S arrest, and decreased the percentage of cells in S phase (Figure 2H-2K). Western blot analysis also found that knockdown of miR-141-3p increased cleaved caspase 3 and decreased cyclin B1, cyclin E1 and CDK2 proteins which correlated with cell apoptosis rate and cell cycle distribution (Supplementary Figure 1A and 1B). And then, we evaluated progression of miR-141-3p in U251 and Ln229 cells which expressed lower levels of miR-141-3p compared to U87 and A172 cells. However, miR-141-3p didn’t promote glioma growth significantly in all experiments as indicated above (Supplementary Figure 2A-2L).

### MiR-141-3p knockdown sensitizes resistant GBM cells to TMZ *in vitro*

**Figure 3:**
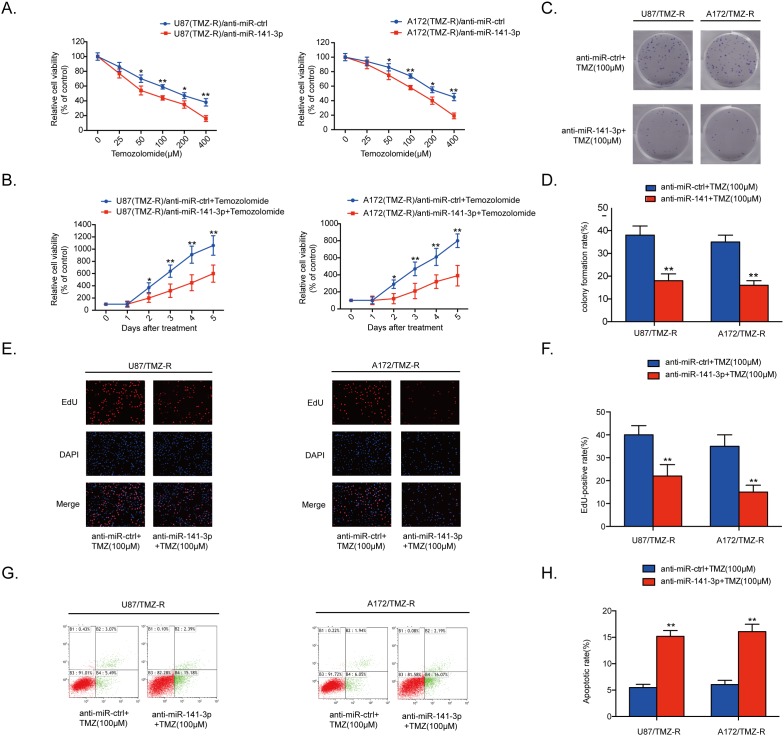
MiR-141-3p knockdown sensitizes resistant GBM cells to TMZ **(A)** Cell proliferation was detected by CCK8 assays in U87/TMZ-R and A172/TMZ-R cells stably expressing anti-miR-ctrl or anti-miR-141-3p and treated with various doses of TMZ. **(B)** Cell proliferation in both U87/TMZ-R and A172/TMZ-R cells stably expressing anti-miR-ctrl or anti-miR-141-3p and treated with 100 μM TMZ was measured every 24 h. **(C** and **D)** Colony formation assays in U87/TMZ-R and A172/TMZ-R stably expressing anti-miR-ctrl or anti-miR-141-3p and treated with 100 μM TMZ. Scale bar: 500 μm. **(E** and **F)** Respective merged images of U87/TMZ-R and A172/TMZ-R cells in EDU stably expressing anti-miR-ctrl or anti-miR-141-3p and treated with 100 μM TMZ after 48 h. Representative images are shown (original magnification, 200×). **(G** and **H)** Apoptosis rates of U87/TMZ-R and A172/TMZ-R cells stably expressing anti-miR-ctrl or anti-miR-141-3p and treated with 100 μM TMZ after 48 h were detected by flow cytometry. All experiments were performed three times using triplicate samples. Average values are indicated with error bars in the histogram. Results are presented as the mean ± S.D. *P<0.05, **P<0.01, and ***P<0.001.

Cell proliferation was detected by CCK8 assays in five glioma cells treated with various doses of TMZ or 100 μM TMZ measured every 24 h. Results showed that TMZ resistance in glioma cells correlated with expression of miR-141-3p (Supplementary Figure 4C). To explore the inhibition of miR-141-3p in chemotherapy response, anti-miR-141-3p or anti-miR-ctrl was transiently transfected into U87/TMZ-resistant (TMZ-R) and A172/TMZ-R cells. qRT-PCR results showed that the relative expression level of miR-141-3p in U87/TMZ-resistant (TMZ-R) and A172/TMZ-R cells treated with anti-miR-141-3p was significantly reduced compared with cells treated with anti-miR-ctrl (P<0.01 for both cell lines; Supplementary Figure 1C). We treated U87/TMZ-resistant (TMZ-R) and A172/TMZ-R cells stably expressing anti-miR-141-3p or anti-miR-ctrl with different concentrations of TMZ (Figure 3A). Knockdown of miR-141-3p in U87/TMZ-R and A172/TMZ-R cells significantly increased chemosensitivity to TMZ treatment, and cell viability was remarkably suppressed by TMZ treatment with an inverse correlation with drug concentrations compared with miR-ctrl group cells. Cell viability in the presence of TMZ (100 μM) was also assayed by CCK8 at different time points, showing that knockdown of miR-141-3p significantly inhibited cell survival of both U87/TMZ-R and A172/TMZ-R cells in the presence of TMZ (Figure 3B). Colony formation and EDU cell image assays were also performed to evaluate the effect of miR-141-3p on cell proliferation in the presence of TMZ (Figure 3C-3E) and yielded results similar to those of the CCK-8 assay. The EDU-positive rate in U87/TMZ-R and A172/TMZ-R cells was lower than that of the control group (P<0.01, Figure 3F). Flow cytometric analysis of apoptosis showed that knockdown of miR-141-3p promoted cell apoptosis in the presence of TMZ and resulted in a higher apoptosis rate (Figure 3G and 3H). Western blot analysis also found that knockdown of miR-141-3p increased cleaved caspase 3 which correlated with cell apoptosis rate (Supplementary Figure 1D).

### p53 is a direct target of miR-141-3p in glioma cells

**Figure 4:**
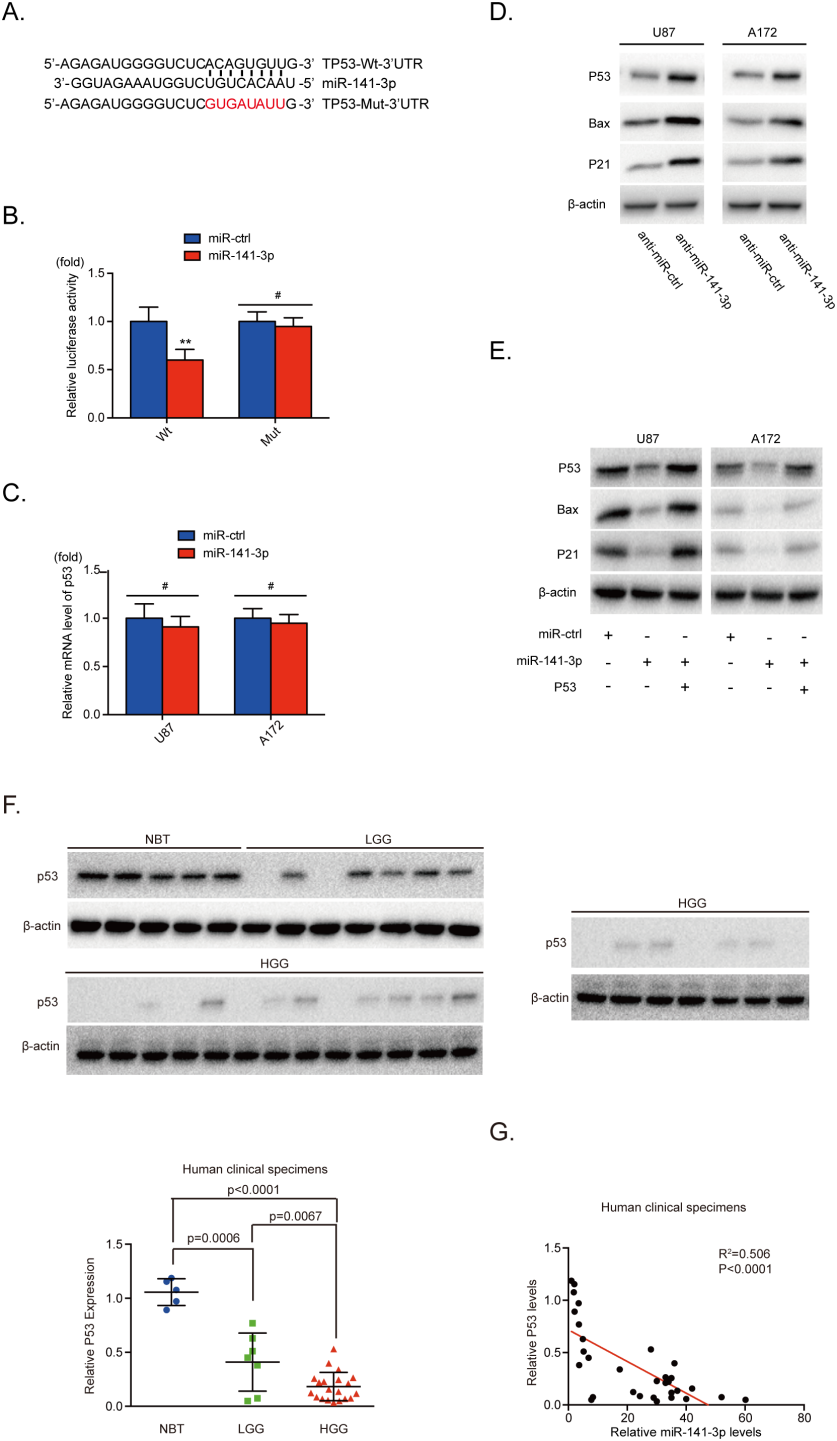
miR-141-3p directly inhibits P53 in glioma cells **(A)** Predicted miR-141-3p target sequence in the 3’UTR of p53 and the mutant containing altered nucleotides in the 3’UTR of p53. **(B)** Luciferase assay of the indicated U87 cells transfected with pmiRNA-Wt-p53-3’UTR, or pmiRNA-Mut-p53-3’UTR reporters and miR-141-3p mimic or miR-ctrl. **(C)** Confirmation of the relative p53 mRNA expression levels in U87 and A172 cells by qRT-PCR. **(D)** Expression of endogenous p53 and its downstream proteins expression in U87 and A172 cells was examined by immunoblotting. **(E)** Forced expression of p53 restored miR-141-3p overexpression. U87 and A172 cells were transfected with miR-141-3p or miR-ctrl, together with or without p53 plasmid. Expression of p53 and its downstream proteins expression in U87 and A172 cells was then examined by immunoblotting. **(F)** Expression levels of p53 in five non-cancerous brain tissues, seven low-grade glioma specimens, and 20 high-grade glioma specimens were determined by immunoblotting. Expression levels were normalized to β-actin. **(G)** Pearson’s correlation analysis was used to determine correlations between p53 protein expression and miR-141-3p levels in specimens as indicated. Results are presented as the mean ± S.D. ^#^P>0.05, *P<0.05, **P<0.01, and ***P<0.001.

To understand the mechanism of miR-141-3p activity in glioblastoma, we employed the bioinformatics analytical tool miRNAWalk 2.0 and TargetScan to identify potential target genes of miR-141-3p and found that the 3′ -UTR of p53 matched the seed sequence of miR-141-3p. To test this combination, 3′ -UTR sequences containing putative binding sites of WT or mutant p53 were constructed in a luciferase report vector in U87 cells (Figure 4A). The relative luciferase activity of Wt-p53 was significantly inhibited (40%) by miR-141-3p mimics whereas that of Mut-p53 was minimally inhibited (P<0.01; Figure 4B). This result indicated that p53 is a direct target of miR-141-3p. Furthermore, we showed that knockdown of miR-141-3p increased protein levels of endogenous p53 and its downstream proteins in both U87 and A172 glioma cells, but mRNA levels showed no notable change (Figure 4C and 4D). This suggests that miR-141-3p inhibits the expression of p53 at the level of translation but not transcription. We also found overexpression of miR-141-3p decreased p53 protein in U251 and Ln229 cells (Supplementary Figure 2N) which the expression of p53 protein were higher, comparing to U87 and A172 cells (Supplementary Figure 2M). To better understand the synergistic effects of p53, U87 and A172 cells were cotransfected with miR-141-3p or miR-ctrl, together with or without p53 plasmid (Figure 4E). We found that transfection of p53 was sufficient to rescue miR-141-3p–induced suppression of p53 and its downstream proteins. In addition, we measured the expression of p53 protein in five non-cancerous brain tissues, seven low-grade glioma tissues, and 20 high-grade glioma tissues. The results shown in Figure 4F and 4G reveal significantly decreased levels of p53 proteins in high-grade glioma or low-grade glioma specimens compared with normal brain tissue. Furthermore, we analyzed the correlation between p53 protein and miR-141-3p levels in all 32 tissues. Pearson’s correlation analysis revealed that p53 levels were inversely correlated with miR-141 expression levels (Pearson’s correlation R^2^=0.506, P<0.0001).

### Reintroduction of p53 could attenuate the oncogenic effect of miR-141-3p

**Figure 5:**
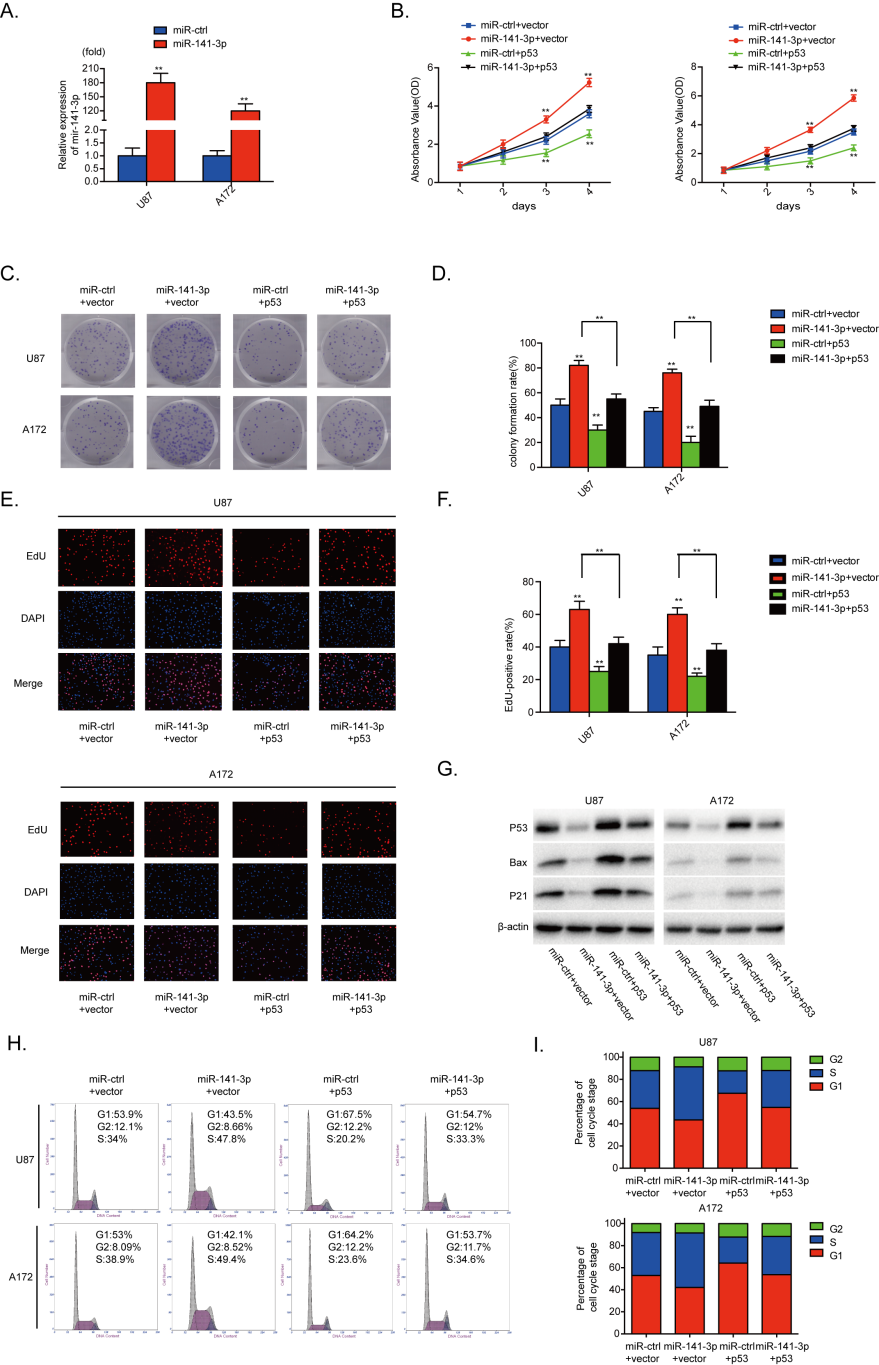
p53 reintroduction reverses the promotional effect of miR-141-3p **(A)** Relative expression of miR-141-3p in U87 and A172 cells was analyzed by qRT-PCR after transfection. **(B)** Cell proliferation was detected by CCK8 assays after U87 and A172 cells transfected with miR-ctrl or miR-141-3p with or without p53 expression plasmid. **(C** and **D)** Colony formation assays in U87 and A172 transfected with miR-ctrl or miR-141-3p with or without p53 expression plasmid. Scale bar: 500 μm. **(E** and **F)** Respective merged images of U87 and A172 cells in EDU transfected with miR-ctrl or miR-141-3p with or without p53 expression plasmid after 48 h. Representative images are shown (original magnification, 200×). **(G)** Downstream proteins of p53 in U87 and A172 cells transfected with miR-ctrl or miR-141-3p with or without p53 expression plasmid were examined by immunoblotting. **(H** and **I)** The cell cycle of U87 and A172 cells transfected with miR-ctrl or miR-141-3p with or without p53 expression plasmid was detected after 48 h by flow cytometry. All experiments were performed three times using triplicate samples. Average values are indicated with error bars in the histogram. Results are presented as the mean ± S.D. *P<0.05, **P<0.01, and ***P<0.001.

U87 and A172 cells were transfected with miR-141-3p (P<0.01, Figure 5A). To evaluate the contribution of p53 to the growth promoting effect of miR-141-3p in glioma cells, we cotransfected human p53 plasmids and miR-141-3p mimics into U87 and A172 cells. As indicated in Figure 5B-5F, 5H and 5I, the effects of miR-141-3p on cell proliferation and cell cycle distribution were inhibited by p53 overexpression. Moreover, increased p53 expression alone markedly inhibited proliferation and promoted G1/S cell cycle arrest in U87 and A172 cells. Cyclin B1, cyclin E1 and CDK2 proteins were also decreased when p53 was overexpressed (Supplementary Figure 3A). Abnormal p53 expression effectively reduced expression of bax and p21, and rescued the increased levels of bax and p21 induced by miR-141-3p mimic in both U87 and A172 cells (Figure 5G). These results further indicate that p53 is a functional target for miR-141-3p in glioma cells.

### Reintroduction of p53 rescues the TMZ resistant effect of miR-141-3p

**Figure 6:**
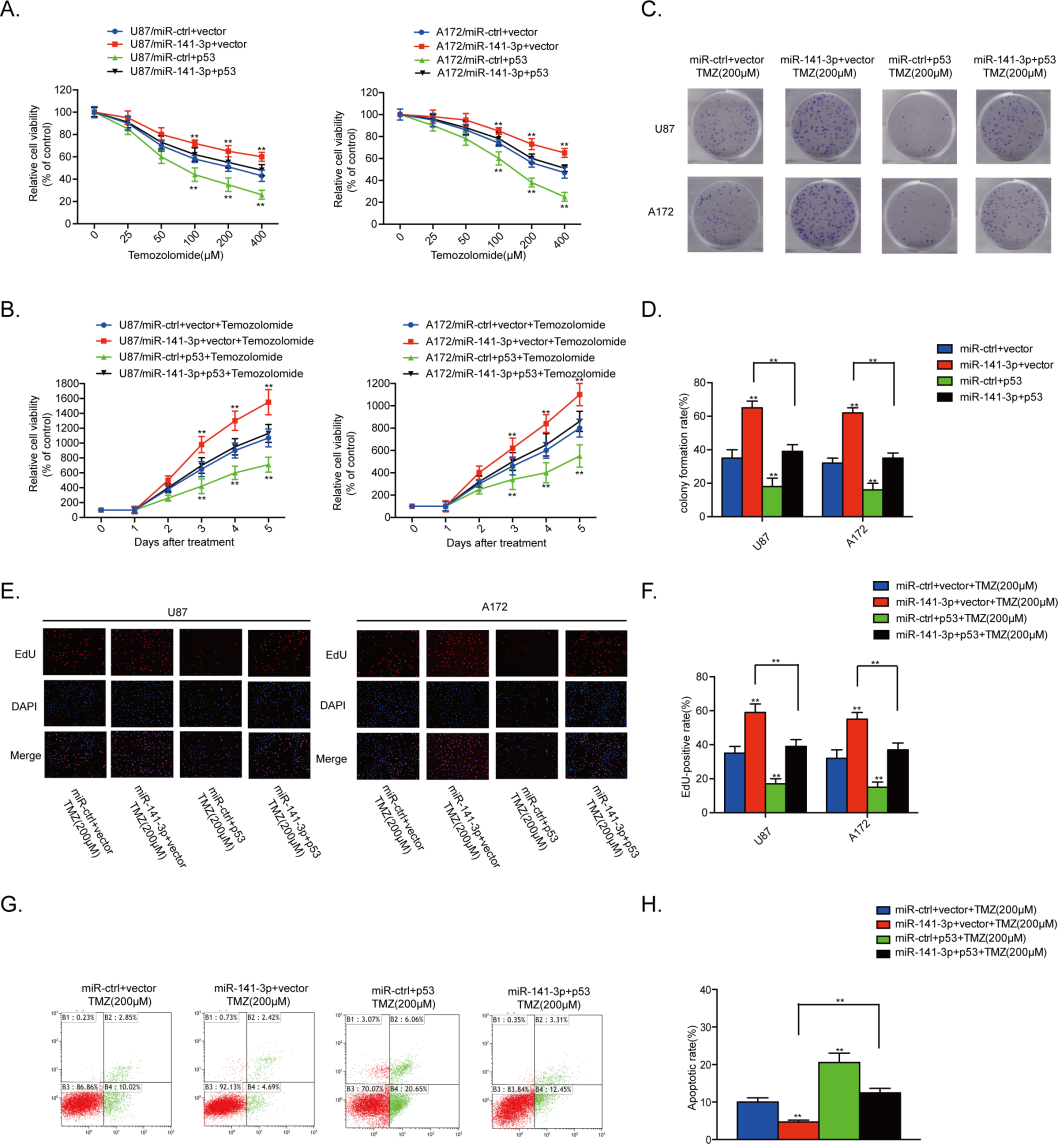
p53 reintroduction rescues the TMZ resistant effect of miR-141-3p **(A)** Cell proliferation was detected by CCK8 assays after U87 and A172 cells were transfected with miR-ctrl or miR-141-3p with or without the p53 expression plasmid and treated with various doses of TMZ. **(B)** Cell proliferation in both U87 and A172 cells transfected with miR-ctrl or miR-141-3p with or without the p53 expression plasmid and treated with 100 μM TMZ was analyzed every 24 h. **(C** and **D)** Colony formation assays in U87 and A172 transfected with miR-ctrl or miR-141-3p with or without the p53 expression plasmid and treated with 100 μM TMZ. Scale bar: 500 μm. **(E** and **F)** Respective merged images of U87 and A172 cells in EDU transfected with miR-ctrl or miR-141-3p with or without the p53 expression plasmid and treated with 100 μM TMZ for 48 h. Representative images are shown (original magnification, 200×). **(G** and **H)** Apoptosis rates of U87 and A172 cells transfected with miR-ctrl or miR-141-3p with or without the p53 expression plasmid and treated with 100 μM TMZ for 48 h were detected by flow cytometry. All experiments were performed three times using triplicate samples, and average values are indicated with error bars in the histogram. Results are presented as the mean ± S.D. *P<0.05, **P<0.01, and ***P<0.001.

We also assessed the contribution of p53 to the TMZ resistance effect of miR-141-3p in glioma cells by co-transfection of human p53 plasmids and miR-141-3p mimics into U87 and A172 cells. The stimulation of proliferation and inhibition of apoptosis by miR-141-3p were suppressed by p53 overexpression in the presence of TMZ. Moreover, increased p53 expression alone markedly suppressed proliferation and promoted cell apoptosis in U87 and A172 cells in the presence of TMZ (Figure 6A-6H). Cleaved caspase 3 protein was also decreased when p53 was overexpressed (Supplementary Figure 3B). These results demonstrated that overexpression of p53 in GBM cells expressing high levels of miR-141-3p reverses TMZ resistance.

### MiR-141-3p knockdown inhibits tumor growth and sensitizes cells to TMZ *in vivo*

**Figure 7:**
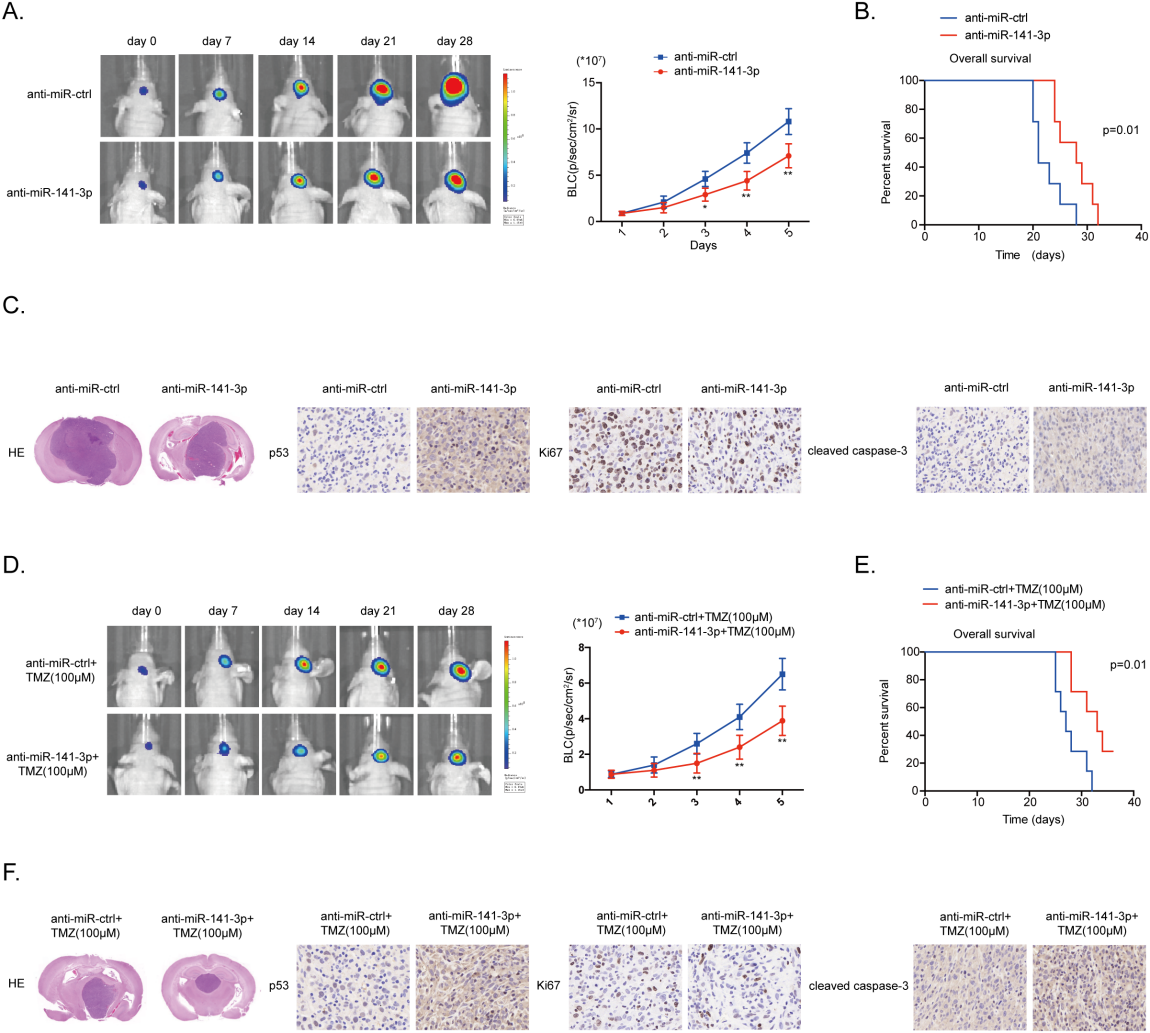
MiR-141-3p knockdown suppresses tumor proliferation and sensitizes TMZ resistant *in vivo* **(A)** U87 cells pre-treated with a lentivirus expressing anti-miR141-3p or anti-miR-ctrl and a lentivirus containing luciferase were implanted in the brains of nude mice. Tumor formation was assessed by bioluminescence imaging. Bioluminescence images were acquired at days 7, 14, 21 and 28 after implantation. **(B)** Overall survival was determined by Kaplan-Meier survival curves. A log-rank test was used to assess the statistical significance of the differences. **(C)** Tissue sections from representative tumors in two groups of U87 cells were stained with Hematoxylin-eosin-saffron. Images show representative immunohistochemical staining for p53, Ki67 and cleaved caspase 3. **(D)** U87/TMZ-R cells stably expressing anti-miR141-3p or anti-miR-ctrl and luciferase, and treated with 100μM TMZ treatments on the days as indicated were implanted in the brains of nude mice. Tumor formation was assessed by bioluminescence imaging. Bioluminescence images were acquired at days 7, 14, 21 and 28 after implantation. **(E)** Overall survival was determined by Kaplan-Meier survival curves. A log-rank test was used to assess the statistical significance of the differences. **(F)** Tissue sections from representative tumors in two groups of U87/TMZ-R cells were stained with Hematoxylin-eosin-saffron. Images show representative immunohistochemical staining for p53, Ki67 and cleaved caspase 3.

To further confirm the effect of miR-141-3p on glioma growth and TMZ resistance we performed an *in vivo* experiment using a U87 xenograft model. Xenografted tumors were induced by U87 glioma cells co-infected with lentiviruses expressing luciferase with anti-miR-141-3p or anti-miR-ctrl, with or without treatment of the mice with TMZ (6 mice per group). In these models, the anti-miR-141-3p group showed a remarkable reduction in intracranial tumor volume compared with the anti-miR-ctrl group. Decreased expression of miR-141-3p significantly inhibited the growth of intracranial tumors at days 14, 21, and 28 after implantation (Figure 7A and 7D). In addition, the anti-miR141-3p group showed significantly longer survival (Figure 7B and 7E). At the termination of the study, tumor volume was remarkably different between the two groups as assessed by staining with hematoxylin and eosin. Moreover, immunohistochemistry showed increased expression of p53, consistent with *in vitro* results (Figure 7C and 7F). Overall, these data indicated that miR-141-3p activates glioma cell growth and sensitizes tumors to TMZ *in vivo*.

## DISCUSSION

MicroRNAs, a class of small regulatory RNAs, have been demonstrated to activate or inhibit a wide variety of oncogenic activities, such as proliferation, cell cycle, cell apoptosis [20] and temozolomide resistance [21]. Dysregulated expression of miRNAs have been observed in various kinds of tumors, including brain tumors such as glioma and its aggressive glioblastoma subtype [22]. Accumulating data indicate that miRNAs are involved in advanced stages of cancer progression and may act as activators or suppressors of tumorigenesis [23]. MiR-141 is a member of the miR-200 family, which also includes miR-200a, miR-200b, miR-200c, miR-141, and miR-429. It has been demonstrated that miR-141 is involved in cancer development, progression and drug resistance regulation [24, 25]. For example, miR-141 is related to ovarian tumorigenesis via targeting of p38a and regulation of the oxidative stress response [26]. Previous studies observed significant upregulation or downregulation of miR-141 in various types of cancers. This differential expression implies that miR-141 activates or inhibits tumors for the initial and developmental stages of cancers [27-29]. In our present study, we found that miR-141-3p was increased in glioblastoma of a higher grade compared with normal brain tissues. Knockdown of miR-141-3p in glioblastoma cells reduced proliferation and induced cell apoptosis, cell cycle arrest, and TMZ resistance. Moreover, decreased expression of miR-141-3p in tumor xenografts in nude mice slowed tumor growth and prolonged the survival of the engrafted mice. We also demonstrated that overexpression of miR-141-3p in glioma cells led to the decreased expression of p21 and bax by directly targeting the 3′-UTR of p53.

The tumor suppressor protein p53 is a pivotal factor in the development of cancer [16, 17]. When DNA damage occurs, p53 is increased by different upstream signals, followed by the activation of various target molecules that participate in the regulation of cell cycle arrest, DNA repair, and apoptosis-related pathways [30].p53 has also been demonstrated to suppress growth, inhibit progression and sensitize Temozolomide (TMZ) in glioma [31-33]. p53 is known to activate a number of effectors, including p21 and bax, and to inhibit cancer cell growth and tumorigenesis [34, 35]. Our results show that miR-141-3p acts as a tumor promoter through various mechanisms, including promotion of tumor cell growth and inhibition of cell apoptosis and induction of cell cycle arrest. Although p53 protein was also decreased when miR-141-3p was transfected into U251 and Ln229 cells, results as indicated above were insignificant. It has been proved that U251 and Ln229 cells were p53 mutation cell lines [36] rather than p53 wild type cell lines U87 and A172 cells [37]. The functions of p53 in U251 and Ln229 cells were lost or changed into other way [38, 39]. So even though miR-141-3p decreased expression of p53 protein, it could not promote U251 and Ln229 cells growth significantly. Moreover, knockdown of miR-141-3p enhanced the chemosensitivity of human glioma cells to TMZ treatment and induced glioma cell apoptosis. Some mechanisms of p53 mutation or not influencing temozolomide resistance has been demonstrated in our another research article and some other research articles which support our results [40, 41]. Of course, p53 is not the only way to affect temozolomide resistance in glioma, some ways which independent of p53 mutation or not has been found as indicated [21]. Both of these can help us study temozolomide resistance in glioma. Thus, we provide a thought that the combination of anti-miR-141-3p plus TMZ could be an effective therapeutic strategy for suppressing the growth of glioma.

In summary, the present study provides new insights into an important relationship between miR-141-3p and tumorigenesis in human glioma. Our findings show that miR-141-3p functions as a tumor promoter by negatively targeting p53. miR-141-3p promotes tumor growth and inhibits cell apoptosis and cell cycle arrest through inhibition of p53 pathways. Interestingly, we demonstrate that miR-141-3p increases the resistance of glioma cells to TMZ treatment. Although miRNA-based therapeutics are still in the initial stages of development, our findings are encouraging and suggest the potential of miR-141-3p as a diagnostic/prognostic marker and novel therapeutic target for glioma.

## MATERIALS AND METHODS

### Cell lines and culture conditions

The human glioma cell lines A172, U87, U251, T98, and Ln229 and normal human astrocytes were purchased from the Chinese Academy of Sciences Cell Bank (Shanghai, China). Temozolomide-resistant cells were generated by repetitive pulse exposure of U87 and A172 glioma cells to TMZ (24 h exposure every 2 weeks). Increasing concentrations of TMZ were applied for 6 months with consideration of the respective EC50 values [42], with selection and culturing of the resistant surviving cell fraction of each passage and consecutive re-exposure to a TMZ pulse after confluent growth. TMZ-resistant cells were regularly challenged with TMZ (33 μM) to sustain the resistant phenotype. Tumor tissue was collected from patients with written patient consent and the following protocols were approved by the Institutional Review Board of the First Affiliated Hospital of Nanjing Medical University. Tissue was obtained from regions of viable tumor. All GBM cell lines were preserved in liquid nitrogen to maintain authenticity and cultured at 37°C in 5% CO_2_ in Dulbecco’s modified Eagle’s medium (DMEM) (Hyclone Laboratories, UT, USA) supplemented with 10% fetal bovine serum (FBS) (Gibco, CA, USA).

### Patient tissue samples

A total of 27 human glioma specimens and 5 normal brain tissues were obtained from The First Affiliated Hospital of Nanjing Medical University. Analysis of human glioma and normal brain tissues was approved by the Research Ethics Committee of Nanjing Medical University (Nanjing, Jiangsu, China) and performed in accordance with the approved guidelines. Informed consent was obtained from all patients included in the study. A total of three large gene expression profiling cohorts of gliomas were used in this study. Data from miRNA expression microarray analysis for 158 gliomas were downloaded from Chinese Glioma Genome Atlas (CGGA) data portal (http://www.cgga.org.cn/portal.php) and 255 samples were downloaded from The Cancer Genome Atlas (TCGA) database (http://tcga-data.nci.nih.gov/). The samples of CGGA comprised 48 astrocytomas (A, WHO Grade II), 13 oligodendrogliomas (O, WHO Grade II), 8 anaplastic astrocytomas (AA, WHO Grade III), 10 anaplastic oligodendrogliomas (AO, WHO Grade III), 15 anaplastic oligoastrocytomas (AOA, WHO Grade III), and 64 GBMs (WHO Grade IV) and of TCGA comprised 10 non-cancerous brains and 245 GBMs (WHO Grade IV).

### Plasmid construction, transfection, and establishment of stable cell lines

Hsa-miR-141-3p mimic and hsa-miR-ctrl were chemically synthesized by Ribobio (Guangzhou, China). Human p53 plasmid was constructed and supplied by Genechem (Shanghai, China) according to the manufacturer’s protocol. Human p53 cDNA was cloned into the pcDNA3 vector to generate pcDNA3-p53 recombinant plasmid (the primer pair sequences used were: Forward:5’-ACGGGCCCTCTAGACTCGAGATGGAGGAGCCGCAGTCAGATCC-3’, Reverse: 5’-GTTCGGGCCCAAGCTTGGTACCTCAGTCTGAGTCAGGCCCTTCTG-3’). A total of 2x10^5^ cells were seeded in 6-well plates with antibiotic-free complete medium. The cells were grown overnight, and then mature miRNA mimics (100 nM) and plasmids (7.5 μg) were transfected into cells using Lipofectamine 2000 Transfection Reagent (Invitrogen, CA, USA) according to the manufacturer’s instructions for 72 h. The lentiviral packaging kit was purchased from Genechem (Shanghai, China). Lentivirus carrying hsa-anti-miR141-3p or hsa-anti-miR-negative control (anti-miR-ctrl) was packaged in human embryonic kidney 293T cells for 48 h and harvested as instructed in the manufacturer’s manual. Stable cell lines were established by infection of U87 and A172 cells which were incubated at a density of 10,000/well seeded in 24-well plates for 24h, and then transfected with 0.5ul lentivirus (1*10^8^ TU/ml), followed by puromycin (1 μg/ml) selection.

### Real-time PCR

RNA was extracted from cell lines and fresh tissues using TRIzol reagent (Life Technologies, CA, USA) following the manufacturer’s protocol. Quantitative real-time PCR was conducted using the ABI StepOne Plus system (Applied Biosystems, CA, USA) with a Bulge-loop™ miRNAqRT-PCR Primer Kit (Ribobio, GuangZhou, China) to detect miR-141-3p level. Primers were purchased from Ribobio (GuangZhou, China). Data were analyzed by the 2^−ΔΔCt^ method and U6 was used as an endogenous control.

### Western blot analysis

Protein extraction and western blotting were performed as described previously [43]. Briefly, cells or tissues were lysed on ice for 30 min in radio-immunoprecipitation assay buffer. The lysates were centrifuged at 12,000 rpm at 4°C for 15 min, the supernatants were collected, and protein concentrations were determined using bicinchoninic acid assay (KenGEN, Jiangsu, China). Equal amounts of protein were separated by 10% SDS-PAGE followed by electrotransfer onto a polyvinylidene difluoride membrane (Thermo Fisher Scientific, MA, USA). Membranes were blocked with 5% nonfat milk for 2 h and incubated with primary antibodies. An electrochemiluminescence detection system (Thermo Fisher Scientific) was used for signal detection. Antibodies against p53(#2527), p21(#2947), bax(#5023), cyclinB1(#12231), cyclinE1(#4129), CDK2(#2546), DAPK1(#3008) and β-actin (#3700) were obtained from Cell Signaling Technology (MA, USA), and cleaved caspase 3(ab2302) obtained from Abcam.

### Cell proliferation assay

Cells in the logarithmic phase of growth were seeded at 3,000 cells per well and cultured in 96-well plates. Cell proliferation was assayed using the Cell-Counting Kit 8 (CCK8, Dojindo Laboratories, Japan) according to the manufacturer’s instructions at the indicated time points. The colony formation assay was performed as described previously [44]. Briefly, 1×10^2^ cells were independently plated onto 6-well tissue culture plates. After 10–20 days, visible colonies were fixed with 4% paraformaldehyde for 20 min and stained with 0.1% crystal violet for 12 h. Colony-forming efficiency was calculated as the number of colonies with diameter >0.5 mm. The 5-ethynyl-2’-deoxyuridine (EdU) proliferation assay was performed with the Molecular Probes EdU-Alexa imaging detection kit (Life Technologies, MA, USA). At 48 h after transfection, cells were incubated with 10 μ M EdU for 2 h, fixed, permeabilized, and stained with both the Alexa-Fluor 594 reaction cocktail for EdU and Hoechst 33342 for cell nuclei, according to the manufacturer's protocol. Samples were imaged under a fluorescent microscope. All assays were repeated at least three times.

### Cell cycle analysis

Transfected cells were harvested, washed with PBS, and fixed with 70% ice-cold ethanol. Fixed cells were resuspended in solution provided in the Cell Cycle Staining Kit (Multi Sciences, Hangzhou, China) and incubated for 30 minutes in the dark before analysis by flow cytometry.

### Analysis of apoptosis

The number of apoptotic cells was counted using an AnnexinV-APC/7-ADD Apoptosis Detection Kit (KeyGEN BioTECH, Jiangsu, China) according to the manufacturer’s protocol. Apoptotic cells were analyzed on a Gallios Flow cytometer (Beckman, CA, USA). The results are presented as the percentage of apoptotic cells relative to the total number of cells.

### Luciferase reporter assay

Wild-type (WT) and mutated putative miR-141-3p seed-matching sites in p53 3’ untranslated regions (UTR) were amplified from human cDNA by PCR and inserted into the Sac I and Hind III restriction enzyme sites of the pmiRNA-Report vector (Genechem, Shanghai, China). U87 cells at a density of 10,000/well were seeded in a 24-well plate and cotransfected with wild-type (WT) or mutated (mut) reporter plasmid (100 ng), miR-141-3p mimic or miR-ctrl (50 nM) as well as internal control Renilla plasmids (100 ng). Luciferase activities were analyzed 24 h after transfection using the Promega Dual Luciferase Reporter Assay System (WI, USA).

### Tumor xenograft studies

All experiments involving mice were performed in The Model Animal Research Center of Nanjing University. Animal experiments were approved by the Animal Management Rule of the Chinese Ministry of Health (documentation 55, 2001) and were performed in accordance with the approved guidelines and experimental protocols of Nanjing Medical University (Nanjing, China). For orthotopic xenograft studies, 2 × 10^5^ GBM cells stably expressing anti-miR-141-3p or anti-miR-ctrl were injected intracranially into the striatum of NOD/SCID mice using a stereotactic device (coordinates: 2 mm anterior, 2 mm lateral, 3 mm depth from the dura). At 1 week, the tumor-bearing mice were given TMZ or placebo by oral gavage (100 μM daily for 5 days per week for three cycles). Tumors were measured by luminescence imaging (IVIS Spectrum, PerkinElmer, USA) every week. The mice were sacrificed upon observation of signs of tumor formation (rough coat, hunching, and weight loss). The brains were extracted and fixed in 10% formalin and then embedded in paraffin for H&E staining and immunochemistry.

### Immunohistochemistry

Immunohistochemical staining of nude mouse xenograft tumor tissues was performed with antibodies against p53 (#2527 Cell Signaling Technology) Ki67 (ab15580 Abcam) as described previously [45] and cleaved caspase 3 (ab2302 Abcam) for apoptosis.

### Statistical analysis

All experiments were performed in triplicate and means and standard error of the mean or standard deviation were subjected to the Student’s t-test for pairwise comparison or ANOVA for multivariate analysis. Kaplan-Meier survival analysis was performed using Graphpad Prism 5 software. Significance level was set at P < 0.05 for all tests.

## SUPPLEMENTARY MATERIALS FIGURES



## Abbreviations

GBM: glioblastoma multiforme
miRNAs: microRNAs
TMZ: temozolomide
CCK-8: Cell Counting Kit-8
EdU: 5-ethynyl-2′-deoxyuridine
