# Supplementary material for: MicroRNA-141-3p promotes glioma cell growth and temozolomide resistance by directly targeting p53
Source: Oncotarget. 2017 Aug 24;8(41):71080–94. doi: 10.18632/oncotarget.20528 (PMC5642619; doi:10.18632/oncotarget.20528)
Supplement: Supplementary file 1 [file oncotarget-08-71080-s001.pdf]

MicroRNA-141-3p promotes glioma cell growth and temozolomide resistance by directly targeting p53

SUPPLEMENTARY MATERIALS

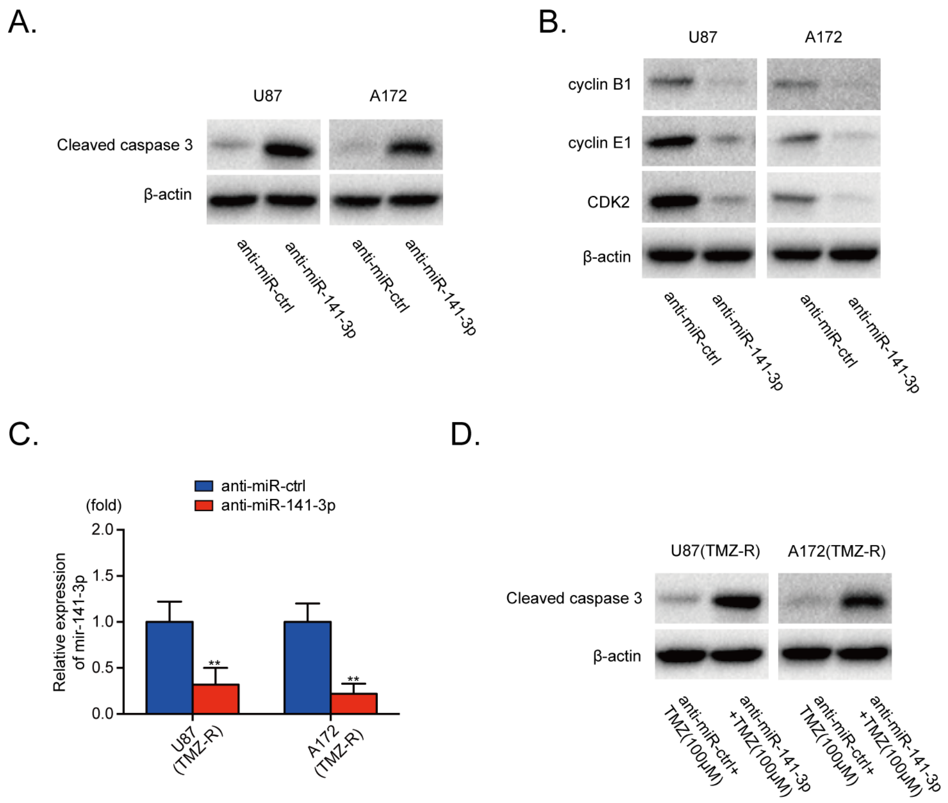

Supplementary Figure 1: MiR-141-3p knockdown inhibits glioma growth and sensitizes resistant GBM cells to TMZ. **(A)** Cleaved caspase 3 protein expression in U87 and A172 cells was examined by immunoblotting. **(B)** Cyclin B1, cyclin E1, and CDK2 protein expression in U87 and A172 cells was examined by immunoblotting. **(C)** Relative expression of miR-141-3p in U87/(TMZ-R) and A172/(TMZ-R) cells was analyzed by qRT-PCR after transfection. **(D)** Cleaved caspase 3 protein expression in U87/(TMZ-R) and A172/(TMZ-R) cells was examined by immunoblotting.

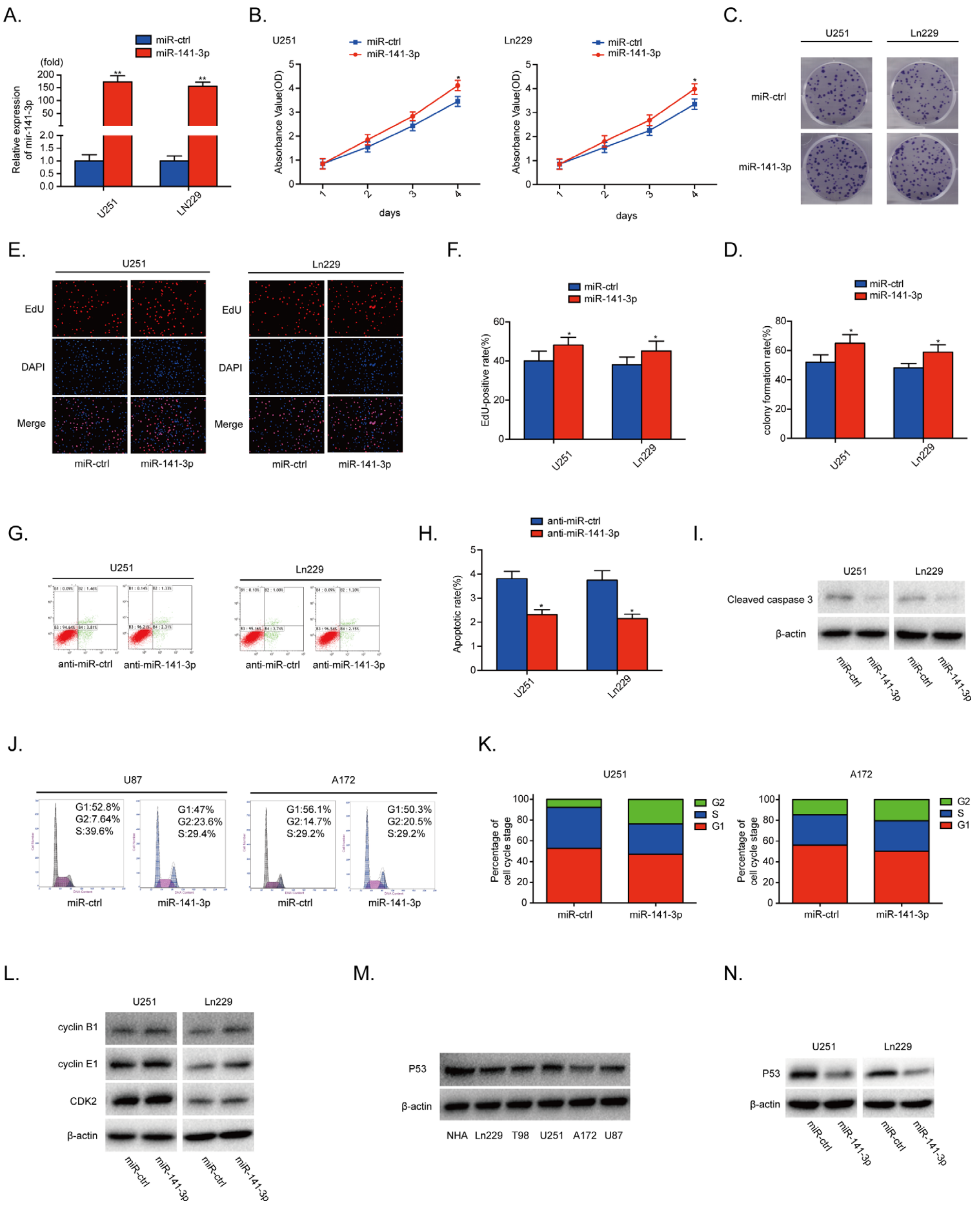

Supplementary Figure 2: MiR-141-3p promotes glioma growth insignificantly in U251 and Ln229 cells. **(A)** Relative expression of miR-141-3p in U251 and Ln229 cells was analyzed by qRT-PCR after transfection. **(B)** Cell proliferation was detected by CCK8 assays in U251 and Ln229 cells transfected with anti-miR-ctrl or anti-miR-141-3p. **(C and D)** Colony formation assays in U251 and Ln229 cells transfected with anti-miR-ctrl or anti-miR-141-3p. Scale bar: 500 μm. **(E and F)** Respective merged images of U251 and Ln229 cells in EDU transfected with anti-miR-ctrl or anti-miR-141-3p after 48 h. Representative images are shown (original magnification, 200×). **(G and H)** Apoptosis rates of U251 and Ln229 cells transfected with anti-miR-ctrl or anti-miR-141-3p after 48 h were detected by flow cytometry. All experiments were performed three times using triplicate samples. Average values are indicated with error bars in the histogram. **(I)** Cleaved caspase 3 protein expression in U251 and Ln229 cells was examined by immunoblotting. **(J and K)** Cell cycles of U251 and Ln229 cells transfected with anti-miR-ctrl or anti-miR-141-3p after 48 h were detected by flow cytometry. All experiments were performed three times using triplicate samples. Average values are indicated with error bars in the histogram. **(L)** Cyclin B1, cyclin E1, and CDK2 protein expression in U251 and Ln229 cells was examined by immunoblotting. **(M)** p53 protein levels in normal human astrocytes and five glioma cell lines. **(N)** Endogenous p53 protein expression in U251 and Ln229 cells was examined by immunoblotting. Results are presented as the mean ± S.D. $*P<0.05$, $**P<0.01$, and $***P<0.001$.

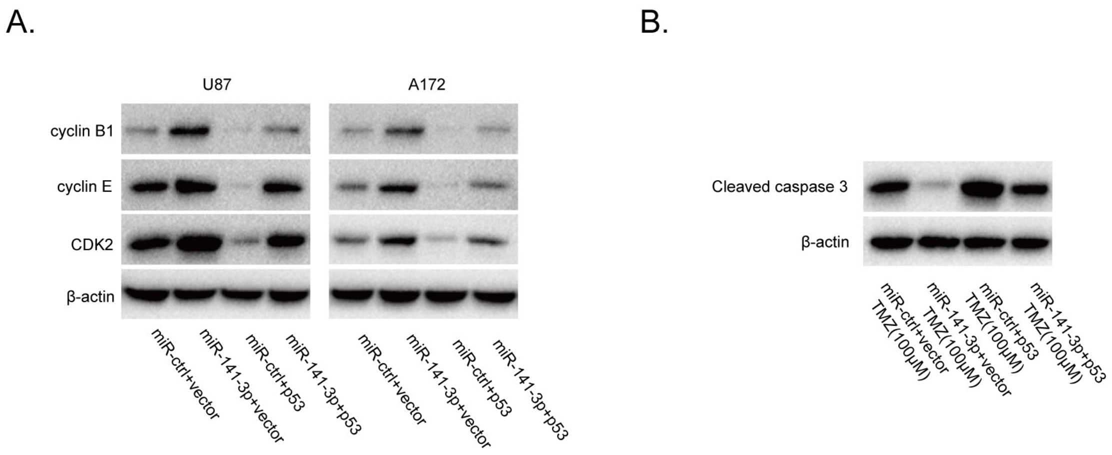

Supplementary Figure 3: p53 reintroduction reverses the promotional and TMZ resistance effects of miR-141-3p. (A) Cyclin B1, cyclin E1, and CDK2 protein expression in U251 and Ln229 cells was examined by immunoblotting. **(B)** Cleaved caspase 3 protein expression in U251 and Ln229 cells was examined by immunoblotting.

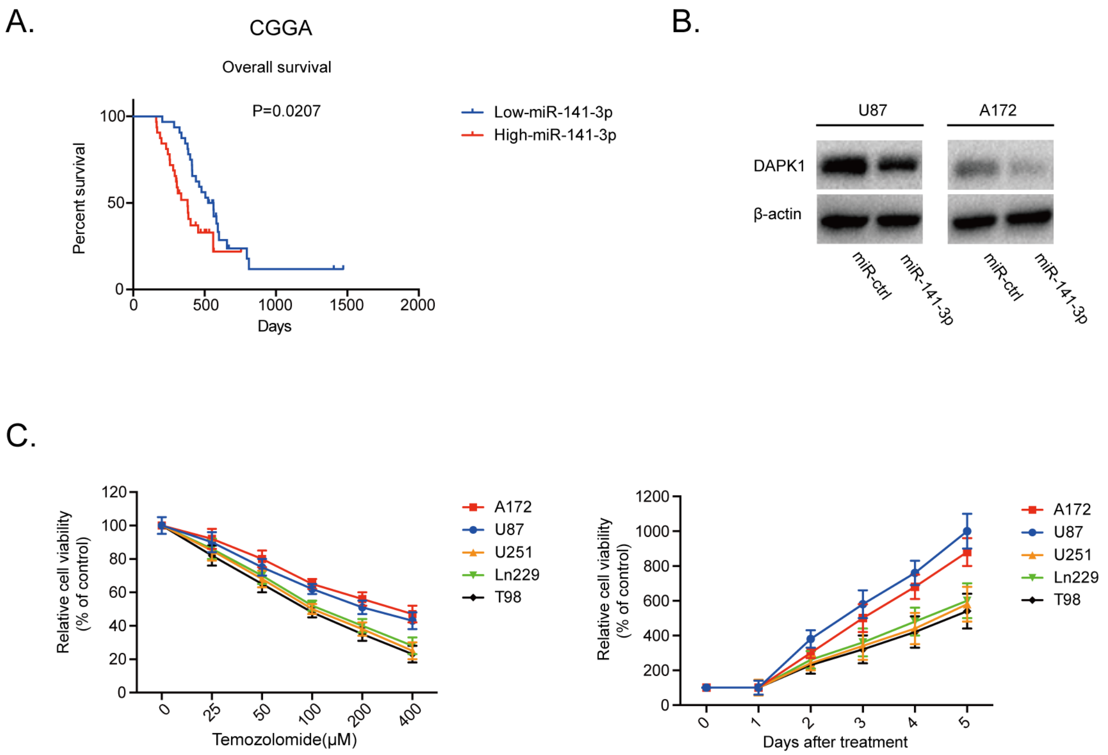

Supplementary Figure 4: MiR-141-3p expression correlates positively with malignant degrees of glioma and TMZ resistance in glioma cells. (A) Correlation between miR-141-3p expression and the overall survival of GBM tissues analyzed by Kaplan-Meier survival curves in the CGGA Public database. A log-rank test was used to assess the statistical significance of differences. **(B)** Expression of DAPK1 in U87 and A172 cells was examined by immunoblotting. **(C)** Cell proliferation was detected by CCK8 assays in five glioma cells treated with various doses of TMZ or 100 μM TMZ measured every 24 h. Results are presented as the mean ± S.D. *P<0.05, **P<0.01, and ***P<0.001.
